# Crystallographic and spectroscopic characterization of (*R*)-*O*-acetyl­mandelic acid

**DOI:** 10.1107/S2056989016008653

**Published:** 2016-06-10

**Authors:** Cady Cirbes, Joseph M. Tanski

**Affiliations:** a Department of Chemistry, Vassar College, Poughkeepsie, NY 12604, USA

**Keywords:** crystal structure, absolute structure, hydrogen bonding, mandelic acid ester derivative

## Abstract

The title compound [systematic name: (*R*)-(−)-2-acet­oxy-2-phenyl­acetic acid], C_10_H_10_O_4_, is a resolved chiral ester derivative of mandelic acid. The compound contains an acetate group and a carb­oxy­lic acid group, which engage in inter­molecular hydrogen bonding, forming chains extending parallel to [001] with a short donor–acceptor hydrogen-bonding distance of 2.676 (2) Å.

## Abstract 2

The title compound is a resolved chiral ester derivative of mandelic acid containing an acetate group and a carb­oxy­lic acid group, which engage in inter­molecular hydrogen bonding, forming chains extending parallel to [001].

## Chemical context   

Chiral, resolved carb­oxy­lic acids have played an important role as chiral NMR shift reagents (Lovely & Wenzel, 2008 ▸; Parker, 1991 ▸). The title compound, (*R*)-(−)-2-acet­oxy-2-phenyl­acetic acid (I), commonly known as (*R*)-*O*-acetyl­mandelic acid, is a chiral, resolved derivative of mandelic acid. The compound may be synthesized by acetyl­ation of the parent α-hy­droxy acid with acetic anhydride in pyridine (Ornelas *et al.*, 2013 ▸). When racemic, resolution of the compound with free amino acids has been demonstrated (Szeleczky *et al.*, 2015 ▸). The title compound has been employed as a chiral NMR shift reagent (Parker, 1991 ▸).
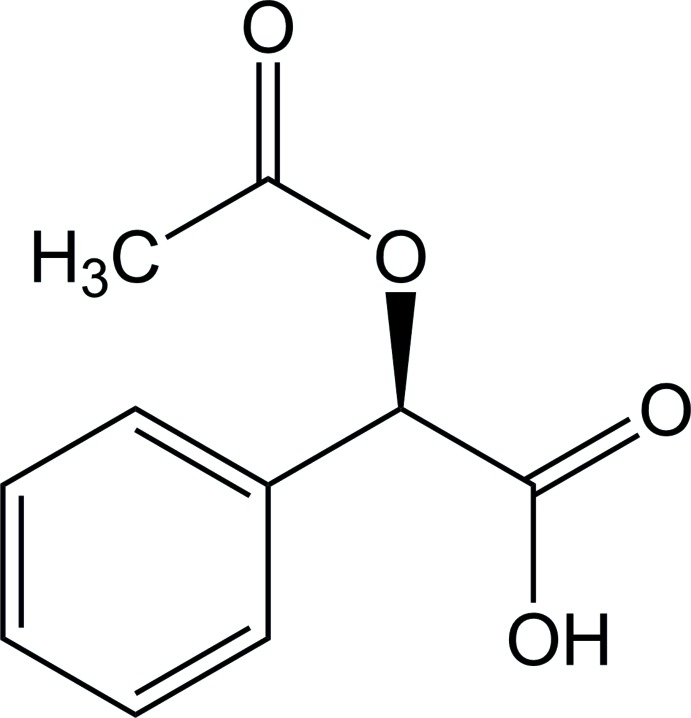


## Structural commentary   

**Figure 1:**
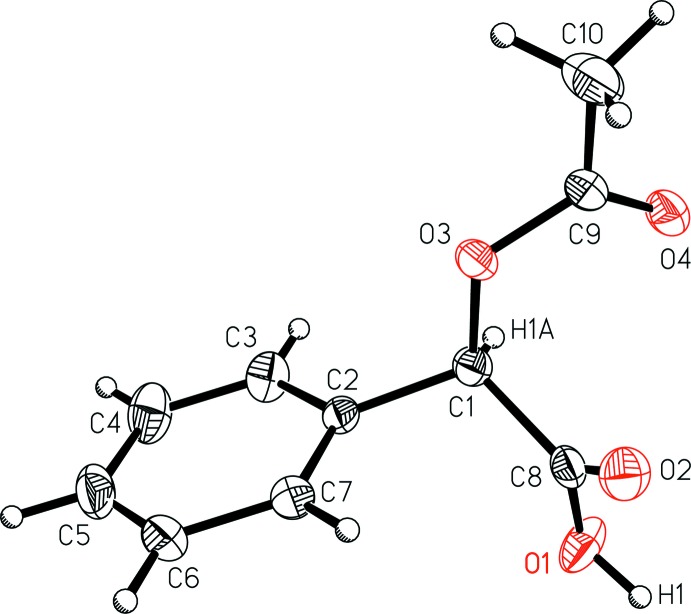
A view of (*R*)-(−)-2-acet­oxy-2-phenyl­acetic acid (I) with the atom-numbering scheme. Displacement ellipsoids are shown at the 50% probability level.

The mol­ecular structure of the title compound (Fig. 1 ▸) shows the *R* confguration about carbon atom C1, and that the mol­ecule does not engage in intra­molecular or pairwise hydrogen bonding. The absolute structure parameters confirm the *R* assignment, with Flack *x* = −0.01 (4) and Hooft *y* = −0.02 (4), calculated with *PLATON* (Spek, 2009 ▸).

## Supra­molecular features   

**Figure 2:**
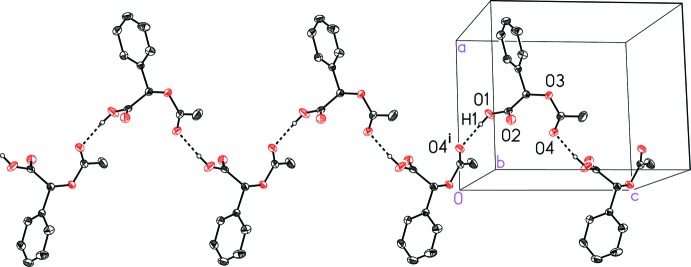
A view of the inter­molecular hydrogen bonding in (*R*)-(−)-2-acet­oxy-2-phenyl­acetic acid (I) that forms a one-dimensional chain. Symmetry code: (i) −*x* + 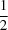, −*y* + 1, *z* − 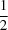.

The mol­ecules pack together in the solid state *via* van der Waals forces and hydrogen bonding between the carb­oxy­lic acid OH group and the carbonyl oxygen atom of the ester on a neighboring mol­ecule, O1—H1⋯O4^i^ [symmetry code (i) −*x* + 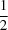, −*y* + 1, *z* − 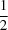] with a donor–acceptor distance of 2.676 (2) Å (Table 1 ▸). These inter­actions create zigzag hydrogen-bonded chains that extend parallel to the *c* axis of the unit cell (Fig. 2 ▸). Notably, there is no face-to-face or edge-to-face π-stacking.

## Database survey   

The Cambridge Structural Database (Groom *et al.*, 2016 ▸) contains several related mandelic acid ester structures. Related structures of resolved mandelic acid esters that differ by the nature of the ester group include (*2S*)-[(*2S*)-2-hy­droxy-2-phenyl­ethano­yloxy]phenyl­acetic acid (Mughal *et al.*, 2004 ▸) and (1*R*,2*R*,3*S*,4*S*)-2-[(*R*)-mandeloxycarbon­yl]bi­cyclo­(2.2.1)heptane-3-carb­oxy­lic acid (Ohtani *et al.*, 1991 ▸). The hydrogen bonding in the former differs from (I), forming an inter­molecular chain with the carb­oxy­lic acid groups further cross-linked by hydrogen bonding of the alcohol moiety with the ester, whereas the latter compound exhibits pairwise dimerization of the carb­oxy­lic acid groups. A related structure with a *tert*-butyl ester and substituents on the phenyl ring, (*S*,*E*)-2-[2-(3-methoxy-3-oxoprop-1-en-1-yl)-4-(trifluoromethyl)phenyl]-2-(pivaloyloxy)acetic acid (Xiao *et al.*, 2016 ▸), exhibits a similar one-dimensional inter­molecular carb­oxy­lic acid OH⋯ester carbonyl hydrogen-bonding motif to that found in the title compound.

## Synthesis and crystallization   

(*R*)-(−)-2-acet­oxy-2-phenyl­acetic acid (99%) was purchased from Aldrich Chemical Company, USA, and was used as received. 

## Analytical data   

^1^H NMR (Bruker Avance 300 MHz, CDCl_3_): δ 2.19 (*s*, 3 H, C*H_3_*), 5.93 (*s*, 1H, C*H*), 7.36–7.42 (*m*, 3 H, C_ar­yl_ *H*), 7.45–7.51 (*m*, 2H, C_ar­yl_ *H*), 11.76 (*br s*, 1H, O*H*). ^13^C NMR (^13^C{^1^H}, 75.5 MHz, CDCl_3_): δ 20.59 (*C*H_3_), 74.02 (*C*H), 127.62 (*C*_ar­yl_H), 128.86 (*C*_ar­yl_H), 129.49 (*C*_ar­yl_H), 132.98 (*C*_ar­yl_), 170.38 (*C*O), 174.55 (*C*O). IR (Thermo Nicolet iS50, ATR, cm^−1^): 3483 (*w*), 3014 (*v b*r, O—H *str*), 2708 (*w*), 2588 (*w*), 1752 (*v s*, C=O *str*), 1686 (*v s*, C=O *str*), 1498 (*w*), 1461 (*w*), 1412 (*m*), 1382 (*s*), 1321 (*m*), 1277 (*s*), 1259 (*s*), 1206 (*s*), 1182 (*s*), 1045 (*s*), 996 (*m*), 967 (*m*), 919 (*m*), 888 (*m*), 767 (s*)*, 734 (*s*), 700 (*s*), 642 (*m*), 616 (*w*), 603 (*w*), 583 (*w*), 525 (*s*), 487 (*w*). GC/MS (Hewlett-Packard MS 5975/GC 7890): *M*-18^+^ = 176 (calc. exact mass 194.06 - water = 176).

## Refinement   

Crystal data, data collection and structure refinement details are summarized in Table 2 ▸. All non-hydrogen atoms were refined anisotropically. Hydrogen atoms on carbon were included in calculated positions and refined using a riding model with C–H = 0.95, 0.98 and 1.00 Å and *U*_iso_(H) = 1.2, 1.5 and 1.2 × *U*_eq_(C) of the aryl, methyl and methine C atoms, respectively. The position of the carb­oxy­lic acid hydrogen atom was found in the difference map and the atom refined semi-freely using a distance restraint *d*(O—H) = 0.84 Å, and *U*_iso_(H) = 1.2*U*_eq_(O).

## Supplementary Material

Crystal structure: contains datablock(s) global, I. DOI: 10.1107/S2056989016008653/pk2580sup1.cif

Structure factors: contains datablock(s) I. DOI: 10.1107/S2056989016008653/pk2580Isup2.hkl

Supporting information file. DOI: 10.1107/S2056989016008653/pk2580Isup3.cml

CCDC reference: 1482445

Additional supporting information: crystallographic information; 3D view; checkCIF report

## Figures and Tables

**Table 1:** Hydrogen-bond geometry (Å, °)

*D*—H⋯*A*	*D*—H	H⋯*A*	*D*⋯*A*	*D*—H⋯*A*
O1—H1⋯O4^i^	0.85 (2)	1.84 (2)	2.6761 (16)	165 (2)

Symmetry code: (i) 
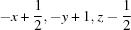
.

**Table 2:** Experimental details

Crystal data	
Chemical formula	C_10_H_10_O_4_
*M*_r_	194.18
Crystal system, space group	Orthorhombic, *P*2_1_2_1_2_1_
Temperature (K)	125
*a*, *b*, *c* (Å)	9.1047 (10), 10.0086 (11), 10.5871 (11)
*V* (Å^3^)	964.75 (18)
*Z*	4
Radiation type	Cu *K*α
μ (mm^−1^)	0.88
Crystal size (mm)	0.26 × 0.26 × 0.17

Data collection	
Diffractometer	Bruker APEXII CCD
Absorption correction	Multi-scan (*SADABS*; Bruker, 2013 ▸)
*T*_min_, *T*_max_	0.74, 0.86
No. of measured, independent and observed [*I* > 2σ(*I*)] reflections	8953, 1698, 1693
*R*_int_	0.030
(sin θ/λ)_max_ (Å^−1^)	0.595

Refinement	
*R*[*F* ^2^ > 2σ(*F* ^2^)], *wR*(*F* ^2^), *S*	0.025, 0.062, 1.10
No. of reflections	1698
No. of parameters	131
No. of restraints	1
H-atom treatment	H atoms treated by a mixture of independent and constrained refinement
Δρ_max_, Δρ_min_ (e Å^−3^)	0.19, −0.19
Absolute structure	Flack *x* determined using 691 quotients [(*I* ^+^)−(*I* ^−^)]/[(*I* ^+^)+(*I* ^−^)] (Parsons *et al.*, 2013 ▸); Hooft *y* calculated with *PLATON* (Spek, 2009 ▸)
Absolute structure parameter	−0.01 (4)

Computer programs: *APEX2* and *SAINT* (Bruker, 2013 ▸), *SHELXT* (Sheldrick, 2015*a* ▸), *SHELXL2014* (Sheldrick, 2015*b* ▸), *SHELXTL* (Sheldrick, 2008 ▸), *OLEX2* (Dolomanov *et al.*, 2009 ▸), *Mercury* (Macrae *et al.*, 2008 ▸) and *PLATON* (Spek, 2009 ▸).

## supplementary crystallographic information

### Crystal data

**Table:**

C_10_H_10_O_4_	*D*_x_ = 1.337 Mg m^−^^3^
*M**_r_* = 194.18	Cu *K*α radiation, λ = 1.54178 Å
Orthorhombic, *P*2_1_2_1_2_1_	Cell parameters from 8300 reflections
*a* = 9.1047 (10) Å	θ = 4.2–66.6°
*b* = 10.0086 (11) Å	µ = 0.88 mm^−^^1^
*c* = 10.5871 (11) Å	*T* = 125 K
*V* = 964.75 (18) Å^3^	Block, colourless
*Z* = 4	0.26 × 0.26 × 0.17 mm
*F*(000) = 408	

### Data collection

**Table:**

Bruker APEXII CCD diffractometer	1698 independent reflections
Radiation source: Cu IuS micro-focus source	1693 reflections with *I* > 2σ(*I*)
Detector resolution: 8.3333 pixels mm^-1^	*R*_int_ = 0.030
φ and ω scans	θ_max_ = 66.6°, θ_min_ = 6.1°
Absorption correction: multi-scan (SADABS; Bruker, 2013)	*h* = −10→10
*T*_min_ = 0.74, *T*_max_ = 0.86	*k* = −11→11
8953 measured reflections	*l* = −12→12

### Refinement

**Table:**

Refinement on *F*^2^	Hydrogen site location: mixed
Least-squares matrix: full	H atoms treated by a mixture of independent and constrained refinement
*R*[*F*^2^ > 2σ(*F*^2^)] = 0.025	*w* = 1/[σ^2^(*F*_o_^2^) + (0.0336*P*)^2^ + 0.1385*P*] where *P* = (*F*_o_^2^ + 2*F*_c_^2^)/3
*wR*(*F*^2^) = 0.062	(Δ/σ)_max_ < 0.001
*S* = 1.10	Δρ_max_ = 0.19 e Å^−^^3^
1698 reflections	Δρ_min_ = −0.19 e Å^−^^3^
131 parameters	Absolute structure: Flack *x* determined using 691 quotients [(*I*^+^)-(*I*^-^)]/[(*I*^+^)+(*I*^-^)] (Parsons *et al.*, 2013); Hooft *y* calculated with *PLATON* (Spek, 2009)
1 restraint	Absolute structure parameter: −0.01 (4)

### Special details

**Table:**

Geometry. All esds (except the esd in the dihedral angle between two l.s. planes) are estimated using the full covariance matrix. The cell esds are taken into account individually in the estimation of esds in distances, angles and torsion angles; correlations between esds in cell parameters are only used when they are defined by crystal symmetry. An approximate (isotropic) treatment of cell esds is used for estimating esds involving l.s. planes.

### Fractional atomic coordinates and isotropic or equivalent isotropic displacement parameters (Å^2^)

**Table:**

	*x*	*y*	*z*	*U*_iso_*/*U*_eq_	
O1	0.42132 (13)	0.46237 (13)	0.08956 (12)	0.0329 (3)	
H1	0.355 (2)	0.479 (2)	0.035 (2)	0.039*	
O2	0.35211 (12)	0.66275 (12)	0.16115 (11)	0.0286 (3)	
O3	0.50941 (12)	0.63319 (11)	0.37499 (9)	0.0235 (3)	
O4	0.29716 (12)	0.53107 (12)	0.42058 (10)	0.0273 (3)	
C1	0.53382 (16)	0.53625 (15)	0.27588 (13)	0.0200 (3)	
H1A	0.5199	0.4438	0.3097	0.024*	
C2	0.69072 (15)	0.55447 (14)	0.23211 (13)	0.0184 (3)	
C3	0.79939 (18)	0.47071 (16)	0.27844 (16)	0.0272 (4)	
H3A	0.7744	0.3988	0.3329	0.033*	
C4	0.94513 (18)	0.49242 (17)	0.2450 (2)	0.0346 (4)	
H4A	1.0198	0.4358	0.2776	0.042*	
C5	0.98206 (18)	0.59621 (18)	0.16417 (17)	0.0326 (4)	
H5A	1.0818	0.6109	0.1418	0.039*	
C6	0.87356 (18)	0.67809 (18)	0.11641 (16)	0.0303 (4)	
H6A	0.8985	0.7482	0.0599	0.036*	
C7	0.72770 (17)	0.65833 (17)	0.15078 (14)	0.0242 (3)	
H7A	0.6534	0.7157	0.1187	0.029*	
C8	0.42388 (15)	0.56275 (15)	0.16993 (14)	0.0201 (3)	
C9	0.38220 (17)	0.62321 (16)	0.43773 (14)	0.0237 (3)	
C10	0.3600 (2)	0.7365 (2)	0.52655 (17)	0.0364 (4)	
H10A	0.4523	0.7555	0.5707	0.055*	
H10B	0.2842	0.7128	0.5883	0.055*	
H10C	0.329	0.8158	0.4792	0.055*	

### Atomic displacement parameters (Å^2^)

**Table:**

	*U*^11^	*U*^22^	*U*^33^	*U*^12^	*U*^13^	*U*^23^
O1	0.0273 (6)	0.0350 (6)	0.0363 (6)	0.0053 (5)	−0.0144 (5)	−0.0139 (5)
O2	0.0270 (6)	0.0289 (6)	0.0298 (6)	0.0060 (5)	−0.0008 (5)	−0.0001 (5)
O3	0.0209 (5)	0.0298 (6)	0.0198 (5)	−0.0061 (4)	0.0051 (4)	−0.0055 (4)
O4	0.0232 (5)	0.0335 (6)	0.0253 (6)	−0.0066 (5)	0.0058 (4)	0.0003 (5)
C1	0.0197 (7)	0.0209 (7)	0.0193 (7)	−0.0032 (6)	0.0012 (6)	−0.0020 (6)
C2	0.0171 (7)	0.0207 (7)	0.0173 (7)	−0.0019 (6)	−0.0001 (5)	−0.0046 (6)
C3	0.0249 (8)	0.0232 (8)	0.0334 (8)	0.0001 (7)	−0.0030 (7)	0.0014 (7)
C4	0.0214 (7)	0.0329 (9)	0.0496 (11)	0.0066 (7)	−0.0035 (8)	−0.0042 (8)
C5	0.0184 (7)	0.0424 (9)	0.0370 (9)	−0.0037 (7)	0.0061 (7)	−0.0119 (8)
C6	0.0276 (8)	0.0394 (9)	0.0238 (8)	−0.0093 (8)	0.0042 (7)	0.0016 (7)
C7	0.0215 (7)	0.0301 (8)	0.0210 (7)	−0.0005 (6)	−0.0010 (6)	0.0037 (6)
C8	0.0149 (7)	0.0237 (7)	0.0216 (7)	−0.0034 (6)	0.0036 (5)	−0.0010 (6)
C9	0.0213 (7)	0.0315 (8)	0.0184 (7)	−0.0036 (7)	0.0033 (6)	0.0021 (6)
C10	0.0390 (10)	0.0389 (10)	0.0311 (9)	−0.0082 (9)	0.0139 (8)	−0.0076 (8)

### Geometric parameters (Å, º)

**Table:**

O1—C8	1.3168 (19)	C3—H3A	0.95
O1—H1	0.852 (19)	C4—C5	1.387 (3)
O2—C8	1.1989 (19)	C4—H4A	0.95
O3—C9	1.3389 (18)	C5—C6	1.380 (3)
O3—C1	1.4463 (17)	C5—H5A	0.95
O4—C9	1.218 (2)	C6—C7	1.391 (2)
C1—C2	1.5128 (19)	C6—H6A	0.95
C1—C8	1.527 (2)	C7—H7A	0.95
C1—H1A	1.0	C9—C10	1.487 (2)
C2—C3	1.386 (2)	C10—H10A	0.98
C2—C7	1.391 (2)	C10—H10B	0.98
C3—C4	1.391 (2)	C10—H10C	0.98
			
C8—O1—H1	107.2 (15)	C4—C5—H5A	120.1
C9—O3—C1	116.28 (11)	C5—C6—C7	120.22 (16)
O3—C1—C2	106.65 (11)	C5—C6—H6A	119.9
O3—C1—C8	108.41 (11)	C7—C6—H6A	119.9
C2—C1—C8	111.91 (12)	C6—C7—C2	119.95 (14)
O3—C1—H1A	109.9	C6—C7—H7A	120.0
C2—C1—H1A	109.9	C2—C7—H7A	120.0
C8—C1—H1A	109.9	O2—C8—O1	125.26 (14)
C3—C2—C7	119.87 (14)	O2—C8—C1	124.01 (14)
C3—C2—C1	119.51 (13)	O1—C8—C1	110.72 (12)
C7—C2—C1	120.54 (13)	O4—C9—O3	122.18 (14)
C2—C3—C4	119.76 (15)	O4—C9—C10	125.84 (14)
C2—C3—H3A	120.1	O3—C9—C10	111.98 (13)
C4—C3—H3A	120.1	C9—C10—H10A	109.5
C5—C4—C3	120.37 (16)	C9—C10—H10B	109.5
C5—C4—H4A	119.8	H10A—C10—H10B	109.5
C3—C4—H4A	119.8	C9—C10—H10C	109.5
C6—C5—C4	119.83 (15)	H10A—C10—H10C	109.5
C6—C5—H5A	120.1	H10B—C10—H10C	109.5
			
C9—O3—C1—C2	−172.13 (12)	C4—C5—C6—C7	1.1 (3)
C9—O3—C1—C8	67.21 (15)	C5—C6—C7—C2	−1.0 (3)
O3—C1—C2—C3	98.10 (15)	C3—C2—C7—C6	−0.2 (2)
C8—C1—C2—C3	−143.51 (14)	C1—C2—C7—C6	176.71 (15)
O3—C1—C2—C7	−78.78 (16)	O3—C1—C8—O2	13.69 (19)
C8—C1—C2—C7	39.62 (18)	C2—C1—C8—O2	−103.65 (16)
C7—C2—C3—C4	1.1 (2)	O3—C1—C8—O1	−167.43 (12)
C1—C2—C3—C4	−175.84 (16)	C2—C1—C8—O1	75.23 (15)
C2—C3—C4—C5	−0.9 (3)	C1—O3—C9—O4	6.3 (2)
C3—C4—C5—C6	−0.2 (3)	C1—O3—C9—C10	−173.19 (13)

### Hydrogen-bond geometry (Å, º)

**Table:**

*D*—H···*A*	*D*—H	H···*A*	*D*···*A*	*D*—H···*A*
O1—H1···O4^i^	0.85 (2)	1.84 (2)	2.6761 (16)	165 (2)
C10—H10*B*···O1^ii^	0.98	2.56	3.312 (2)	133

Symmetry codes: (i) −*x*+1/2, −*y*+1, *z*−1/2; (ii) −*x*+1/2, −*y*+1, *z*+1/2.
